# Using exosomal miRNAs extracted from porcine follicular fluid to investigate their role in oocyte development

**DOI:** 10.1186/s12917-020-02711-x

**Published:** 2020-12-14

**Authors:** Junhe Hu, Jinyi Dong, Zhi Zeng, Juan Wu, Xiansheng Tan, Tao Tang, Jiao Yan, Chenzhong Jin

**Affiliations:** College of Agriculture and Biotechnology, Hunan University of Humanities, Science and Technology, Road Dingxing 7#, Loudi City, 417000 HuNan Province China

**Keywords:** Exosomes, Porcine follicular fluid (PFF), Oocyte maturation, Follicular development, miRNA

## Abstract

**Background:**

Follicular development is crucial to normal oocyte maturation, with follicular size closely related to oocyte maturation. To better understand the molecular mechanisms behind porcine oocyte maturation, we obtained exosomal miRNA from porcine follicular fluid (PFF). These miRNA samples were then sequenced and analyzed regarding their different follicular sizes, as described in the methods section.

**Results:**

First, these results showed that this process successfully isolated PFF exosomes. Nearly all valid reads from the PFF exosomal sequencing data were successfully mapped to the porcine genome database. Second, we used hierarchical clustering methods to determine that significantly expressed miRNAs were clustered into A, B, C, and D groups in our heatmap according to different follicle sizes. These results allowed for the targeting of potential mRNAs genes related to porcine oocyte development. Third, we chose ten, significantly expressed miRNAs and predicted their target genes for further GO analysis. These results showed that the expression levels of neurotransmitter secretion genes were greatly changed, as were many target genes involved in the regulation of FSH secretion. Notably, these are genes that are very closely related to oocyte maturation in growing follicles. We then used pathway analysis for these targeted genes based on the originally selected ten miRNAs. Results indicated that the pathways were mainly related to the biosynthesis of TGF-beta and its signaling pathway, which are very closely related to reproductive system functions.

**Conclusions:**

Finally, these exosomal miRNAs obtained from PFF may provide a valuable addition to our understanding of the mechanism of porcine oocyte maturation. It is also likely that these exosomal miRNAs could function as molecular biomarkers to choose high-quality oocytes and allow for in vitro porcine embryo production.

## Background

It is well known that oocyte maturation is strongly related to follicular growth and development [1]. As the follicles grow, their size is closely accompanied by increased oocyte diameter, indicating the ever-progressing maturation status of the oocyte. Many past studies have shown that oocyte quality is highly correlated with oocyte diameter and later embryo development [1–4]. Moreover, some bovine oocyte maturation increases the volume of follicular fluid, owing to the larger follicle size. This may improve overall oocyte nuclear and cytoplasmic maturation when compared to follicles with smaller sizes and lower fluid volumes [5]. Given this, follicular size is a good predictor of oocyte quality and potential development and is a useful tool to better understand the molecular mechanisms of oocyte maturation.

Exosomes (EXs) are small (30–150 nm), cell-derived vesicles that are present in many-and perhaps all-eukaryotic fluids, including human follicular fluid, blood, and cultured cellular medium. Moreover, past work has found that EXs contain a large number of biologically active molecular substances, such as proteins, mRNAs, and miRNAs. These substances exert myriad biological functions and are released from the cells and into the extracellular environment through budding and fission from the plasma membrane [6].

Structurally, EXs are formed by the invagination of multivesicular bodies in the lumen, which then fuse to the plasma membrane and release their contents to the cells in a manner that extracellularly transports the carrie [7]. In the cell and under normal physiological conditions, EXs are separated from the vesicular structure in the endoplasmic reticulum. Transport to the plasma membrane allows extracellular release and for EXs to exert regulatory control over normal physiological functions [8]. Given this, EXs likely play an important role in regulating oocyte physiological functions by transporting materials. In turn, these have targeted, cell signaling functions by containing a variety of RNAs [9].

Past work has reported that EXs exist under normal physiological conditions in horse [10] as well as human [11] follicular fluid. EXs also exist in bovine follicular fluid under conditions of reduced milk production [12]. Additional work has shown that these extracellular miRNAs play a role in regulating external environmental influences through the protective devices packaged as EXs [13]. Collectively, these molecules play essential roles across a wide range of physiological processes, some of which play a critical role in reproduction functions (e.g., follicular development and oocyte maturation) [14]. More specifically, past work has shown that miRNAs exist in porcine follicular fluids [12]; however, there is only a few limited reports about non-coding exosomal RNA transcripts in PFF to affect oocyte development.

Given this past work and our current understanding, it is very likely that EXs play a vital role in the relationship and regulation of associated cells in the follicular fluid during oocyte growth and development, which is achieved by their packaged biological materials, including miRNAs. Although our knowledge regarding oocyte quality and development has significantly improved in recent years, the molecular mechanisms that determine and regulate oocyte development remain unclear. Given this, we sought to conduct a study to compare the expression of exosomal, non-coding RNA transcripts from the PFF of different-sized follicles.

The results of this study substantially revise our understanding of the content of porcine follicular fluid exosomes and how they influence oocyte maturation by interactions with follicular somatic cells and cumulus cells around the oocytes. Ultimately, this work lays the foundation for future investigations regarding the role and mechanistic importance of PFF exosomal miRNAs in oocyte development. Therefore, the objective of this study was to identify and analyze the transcriptomic profiles of PFF exosomes derived from different-sized follicles using RNA high-throughput sequencing technology.

## Methods

### Porcine follicular fluid samples collection

All experimental porcine ovaries were collected from a local abattoir (usually 60 porcine ovaries each time collected from QinYangMuYe Limited Responsibility Company, LouDi city, HuNan Province, China) and transported to our lab within 2 h of slaughter in a thermo-flask containing penicillin and streptomycin. All experiments were conducted in accordance with the Institutional Ethics Committee of Hunan University of Humanities, Science and Technology. Ovaries were washed at least three times with physiological saline solution and porcine follicular fluid was collected by separately aspirating with a 23-gauge needle attached to a 5 mL syringe from small follicles (small follicles only; < 3 mm [group A as control group] and 3–5 mm [group B]) or a 20-gauge needle attached to a 10 mL syringe from large follicles (large follicles only; 5–8 mm [group C] and > 8 mm [group D], which each group has three duplicate samples shown as following: A3, A5 and A6 in group A; B3, B5 and B6 in group B; C3, C4 and C5 in group C; D1, D5, and D6 in group D. Follicle diameter was measured as previously reported [15]. All follicular fluid samples were centrifuged at 1300×g for 15 min to remove any cells; at least 10 mL suspended follicular fluid was stored at − 80 °C until later experiments, and precipitated blood and the other material was s discarded.

### Exosome purification and characterization

Porcine Follicular Fluid exosomes were purified and characterized according to previously published protocols with slight modifications [16]. Briefly, the isolation method used was as follows: Pooled 12 samples (total volume 15 mL) was centrifuged at 1300×g for 15 min at 4 °C to separate and isolate any debris. The supernatant was then transferred to a separate 15 mL ultracentrifuge tubes, after which it was ultracentrifuged at 16,500×g for 30 min at 4 °C. The resulting supernatant was then filtered through a 0.2 mm syringe filter to obtain the exosome-containing medium. Finally, exosomes were pelleted after ultracentrifugation at 120,000×g for 70 min at 4 °C and stored at − 80 °C until later analysis.

Exosome (EX) pellets were suspended in PBS for electron microscopy (EM) analysis. First, resuspended EXs samples (5–10 μL per sample) were added to the copper mesh and precipitated for 3 min. Filter paper was used to absorb any volatile liquid from the edge. After rinsing with PBS, negative dying of phosphotungstic acid was performed. The sample was then dried at room temperature (2 min) for subsequent imaging (EM operating voltage 80–120 kv).

### Construction of exosomal small RNA library

Exosome pellets were firstly resuspended in Trizol (Thermo Fisher Technology (China) Co., Ltd., Shanghai, China) for further RNA isolation. A small EX RNA library was then constructed using total RNA from 15 mL pooled follicular fluid. Small RNA cloning, sequencing, and analysis were conducted as previously described using QIAseq® miRNA Library Kit [17]. Briefly, 20 ng of total RNA underwent rRNA depletion and DNase digestion using a NEBNext rRNA Depletion Kit (New England Biolabs (Beijing) LTD, Beijing, China) according to the manufacturer’s protocol. A nucleoMag NGS Clean-up and Size Select Kit (Thermo Fisher Technology (China) Co.,Ltd., Shanghai, China,) was used for sample purification and library size selection. RNA samples were purified, fragmented at 94 °C for 15 min, and primed with random primers. Samples were then converted to double-stranded cDNA, purified, and adapters were ligated to the 3′ and 5′ ends. The cDNA samples were amplified by PCR (14 cycles) using indexing forward primers and a universal reverse primer. After PCR amplification, RNA libraries were purified and quality control of RNA was conducted using an Agilent 2100 Bio-analyzer (Agilent Technologies (China) Co., Ltd., Beijing, China).

### RNA-Seq data analysis of EXs obtained from porcine follicular fluids

Sequencing adaptors and low-complexity reads were removed in an initial data filtering step. The quality of the reads was then checked using the FASTQC program freely from the internet. Reads were then aligned against a swine reference genome (Sscrofa11.1, January 2017), which was downloaded from the Swine Genome Sequencing Consortium project (http://www.ensembl.org/) and used for all subsequent bioinformatic analyses. Finally, we applied a DEBseq-counts algorithm to filter the differentially expressed genes after significant analysis and FDR analysis under the following criteria: Log2FC > 1 or FDR < 0.05. This was done to select for any significantly expressed genes for additional research. Functional annotation was performed using the Database for Annotation, Visualization and Integrated Discovery (DAVID) v6.8 (http://david.ncifcrf.gov). Pathway analysis was conducted using the download annotation data from KEGG (http://www.genome.jp/kegg/) and Cytoscape (https://cytoscape.org/) software.

### GO and pathway analyses

Based on the hierarchical structure of GO, the mutual control and subordinate relationships between all GO were organized into a database. By constructing a functional relationship network, it is easy to summarize the functional groups affected by the experiment and the intrinsic subordinate relationships of the significant functions. Functional regulation analysis was performed using the significant GO-Term (*p* < 0.01) in GO-Analysis by differential genes to construct a functional regulatory network. GO classification can describe the function of genes from various aspects, and can be divided into three main groups: Biological Process (BP), Molecular Function (MF), and Cellular Component. The selected differentially expressed genes were annotated using this pathway approach and based on the KEGG database; all pathway terms in which the differentially expressed genes and their target genes were then obtained.

### Statistical analysis

Both the back-spliced junction reads and the linear mapped reads were combined and scaled to reads per kilobase per million mapped reads (RPKM) to quantify miRNA expression levels. Differences in these miRNA expression profiles between groups A, B, C, and D were analyzed using Student’s t-test. *P* < 0.05 was considered statistically significant.

## Results

### Exosome isolation and quality control of the RNA-sequence data

**Fig. 1:**
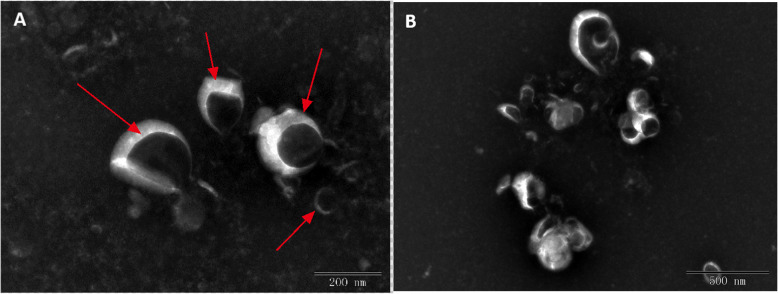
PFF exosome confirmation using transmission electron microscopy (TEM). **a**. TEM of exosomes imaged using 200 nm microscope rulers. Exosomes present a cup-shaped morphology (indicated as red arrow). **b**. TEM of exosomes imaged using 500 nm size microscope rulers

Porcine Follicular Fluid exosomes were successfully separated and further verified according to the above methods. These exosomes were also analyzed using transmission electron microscopy (JEM-1200EX) with an operating voltage of 80–120 kv (Fig. 1a-b). Exosome pellets were resuspended in phosphate-buffered saline (PBS) for subsequent electron microscopy analysis to further verify their characterization according to previously published results [18]. FastQC software (https://www.bioinformatics.babraham.ac.uk/projects/fastqc/) was used to perform a full evaluation of the sequenced data, including length distribution, quality score, and GC content. The quality control of the raw sequencing data sought to provide a quick impression of whether the data had any problems. This step was necessary to ensure awareness of any problems prior to further analysis according to a previously published method [19].

### miRNAs with significant expression and their heatmap analysis

RNA sequencing and analysis were conducted using 16 samples: Group A: A3, A5, and A6; Group B: B3, B5, and B6; Group C: C3, C4, and C5; Group D: D1, D5, and D6. Three replicates for each sample were used per group. Samples were prepared by collecting different size follicles according to the above methods. On average, the total number of valid reads obtained from PFF exosomes was approximately 6.50 million for group A, 4.30 million for group B, 6.90 million for group C, and 9.30 million for group D (Table 1). Overall, approximately 99% of all valid reads were from groups A-D. From these and as indicated in Table 1, all 12 samples were successfully mapped to the porcine non-coding RNA database for further research.

**Table 1:** The sequencing data obtained after sequencing of different experimental groups were compared with the existing swine genome statistics

Statistics	A1	A2	A3	B1	B2	B3	C1	C2	C3	D1	D2	D3
	A3 Result	A5 Result	A6 Result	B3 Result	B5 Result	B6 Result	C3 Result	C4 Result	C5 Result	D1 Result	D5 Result	D6 Result
All	9.74E+ 06	8.28E+ 06	1.10E+ 07	5.51E+ 06	7.98E+ 06	7.30E+ 06	6.31E+ 06	5.84E+ 06	8.49E+ 06	1.01E+ 07	7.97E+ 06	1.00E+ 07
UnMapped	24,908	14,627	21,357	18,332	29,536	18,978	23,296	15,669	31,405	33,521	22,323	90,820
Mapped	9.72E+ 06	8.26E+ 06	1.10E+ 07	5.49E+ 06	7.95E+ 06	7.29E+ 06	6.29E+ 06	5.83E+ 06	8.46E+ 06	1.01E+ 07	7.95E+ 06	9.91E+ 06
MappedRate	0.997	0.998	0.998	0.997	0.996	0.997	0.996	0.997	0.996	0.997	0.997	0.991
UniqueMapped	420,828	250,095	393,684	390,443	310,342	397,850	380,636	381,043	364,489	325,603	293,976	475,844
UniqueMappedRate	0.043	0.03	0.036	0.071	0.039	0.054	0.06	0.065	0.043	0.032	0.037	0.048
RepeatMapped	9.30E+ 06	8.01E+ 06	1.06E+ 07	5.10E+ 06	7.64E+ 06	6.89E+ 06	5.91E+ 06	5.45E+ 06	8.09E+ 06	9.74E+ 06	7.65E+ 06	9.43E+ 06
AllBase	2.25E+ 08	1.80E+ 08	2.35E+ 08	1.22E+ 08	1.77E+ 08	1.54E+ 08	1.42E+ 08	1.24E+ 08	1.95E+ 08	2.54E+ 08	1.88E+ 08	2.15E+ 08
UnMappedBase	712,918	416,373	602,089	516,508	811,214	535,743	657,339	437,772	894,895	959,599	632,346	2,564,625
MappedBase	2.24E+ 08	1.80E+ 08	2.34E+ 08	1.22E+ 08	1.76E+ 08	1.53E+ 08	1.41E+ 08	1.23E+ 08	1.94E+ 08	2.53E+ 08	1.87E+ 08	2.13E+ 08
UniqueMappedBase	8.82E+ 06	4.93E+ 06	7.57E+ 06	7.67E+ 06	6.15E+ 06	7.53E+ 06	7.41E+ 06	7.13E+ 06	7.05E+ 06	6.74E+ 06	5.98E+ 06	9.61E+ 06
RepeatMappedBase	2.16E+ 08	1.75E+ 08	2.27E+ 08	1.14E+ 08	1.70E+ 08	1.46E+ 08	1.34E+ 08	1.16E+ 08	1.87E+ 08	2.46E+ 08	1.81E+ 08	2.03E+ 08

**Fig. 2:**
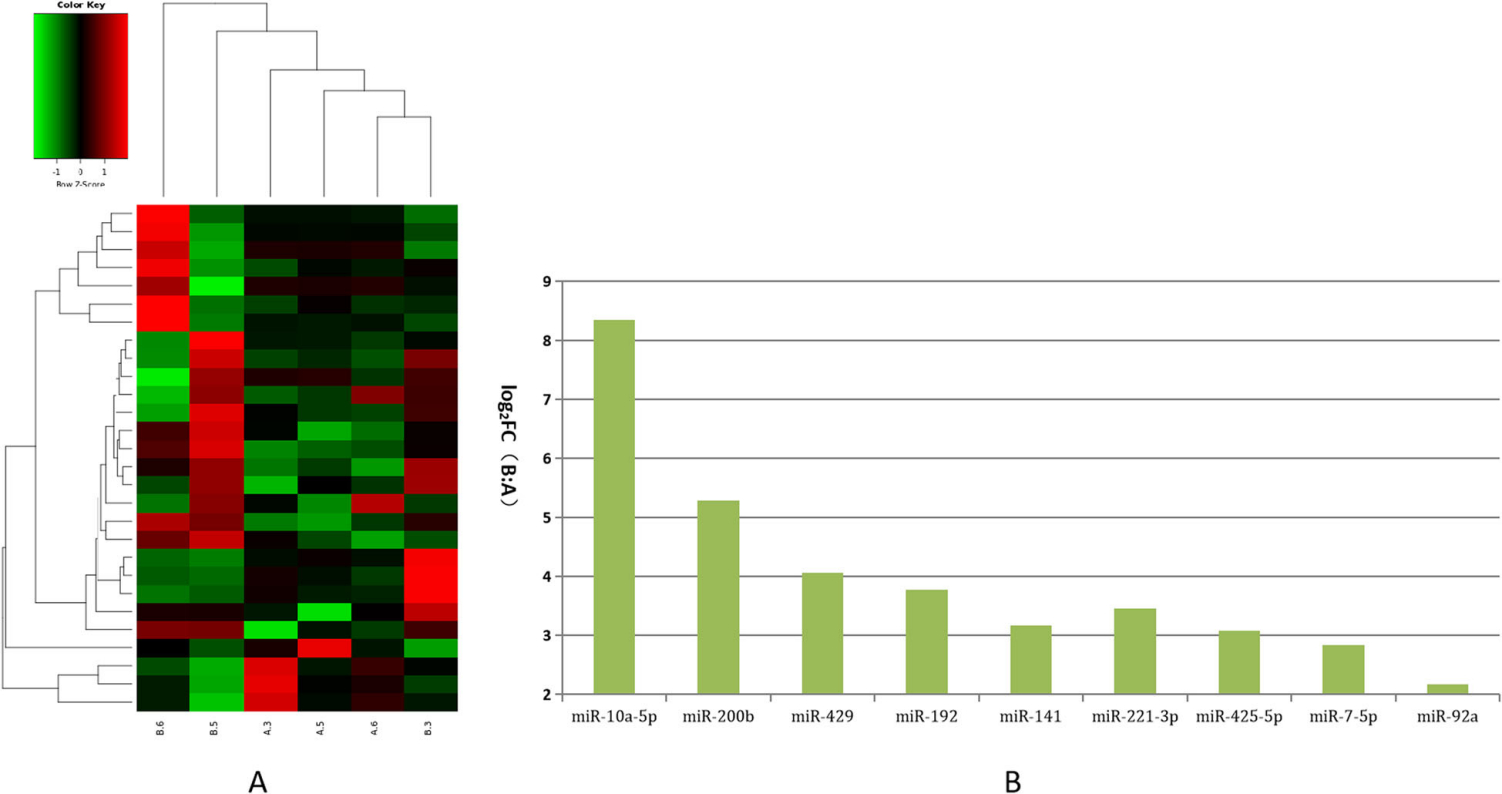
Comparison of differentially expressed miRNAs between groups A and B. **a**. Heatmap of clustered genes shows significantly different between groups A and B. **b**. The most significantly and highly expressed miRNA genes when comparing groups B and A. Y-axis indicates log2(Fold Changes, FC) of miRNA expression of group B versus A

**Fig. 3:**
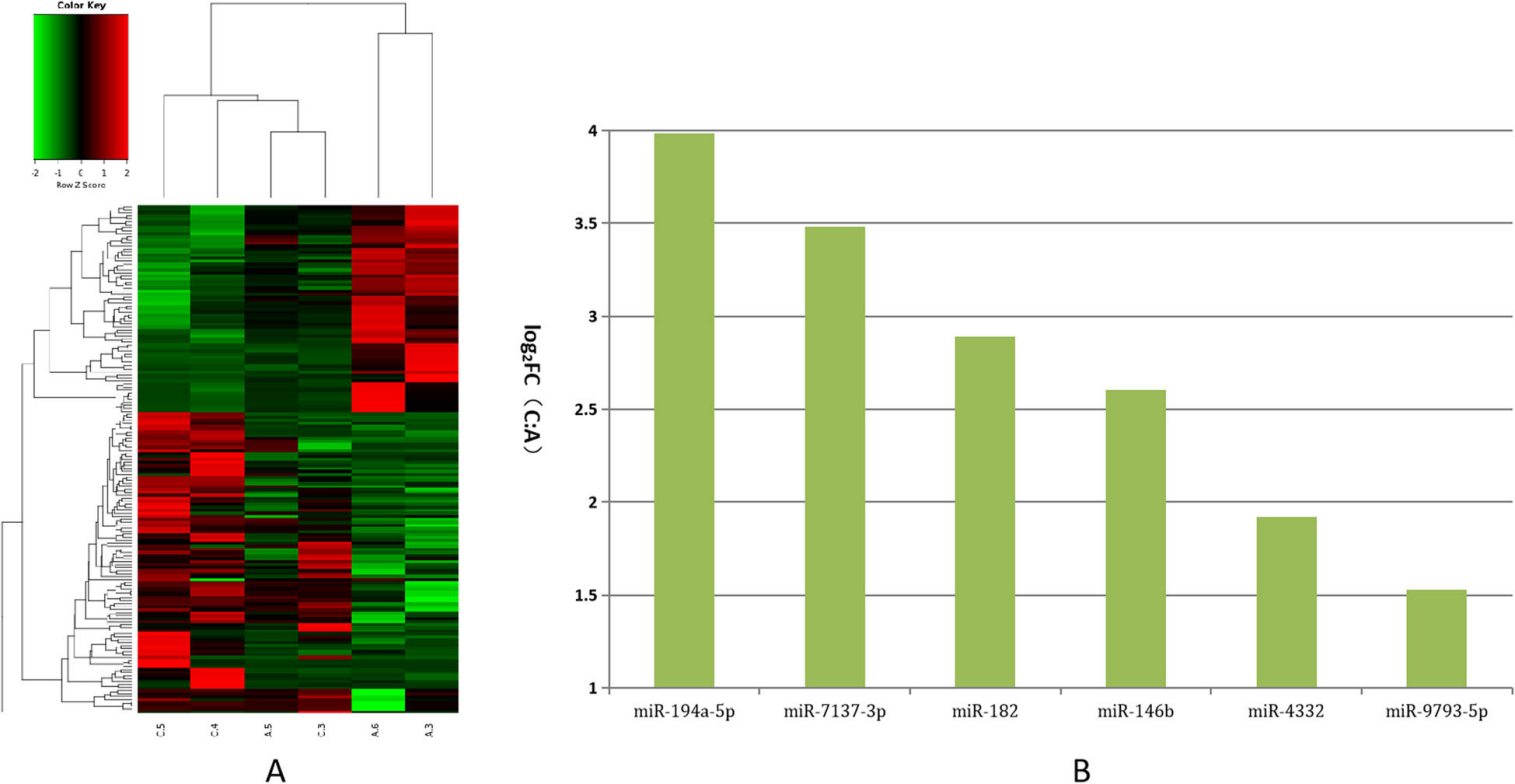
Comparison of most differentially expressed miRNAs between groups A and C. **a**. Heatmap of clustered genes indicates significantly different between groups A and C. **b**. Most significantly expressed miRNA genes between groups A and C. Y-axis indicates log2(Fold Changes, FC) of miRNA expression of group C versus A

**Fig. 4:**
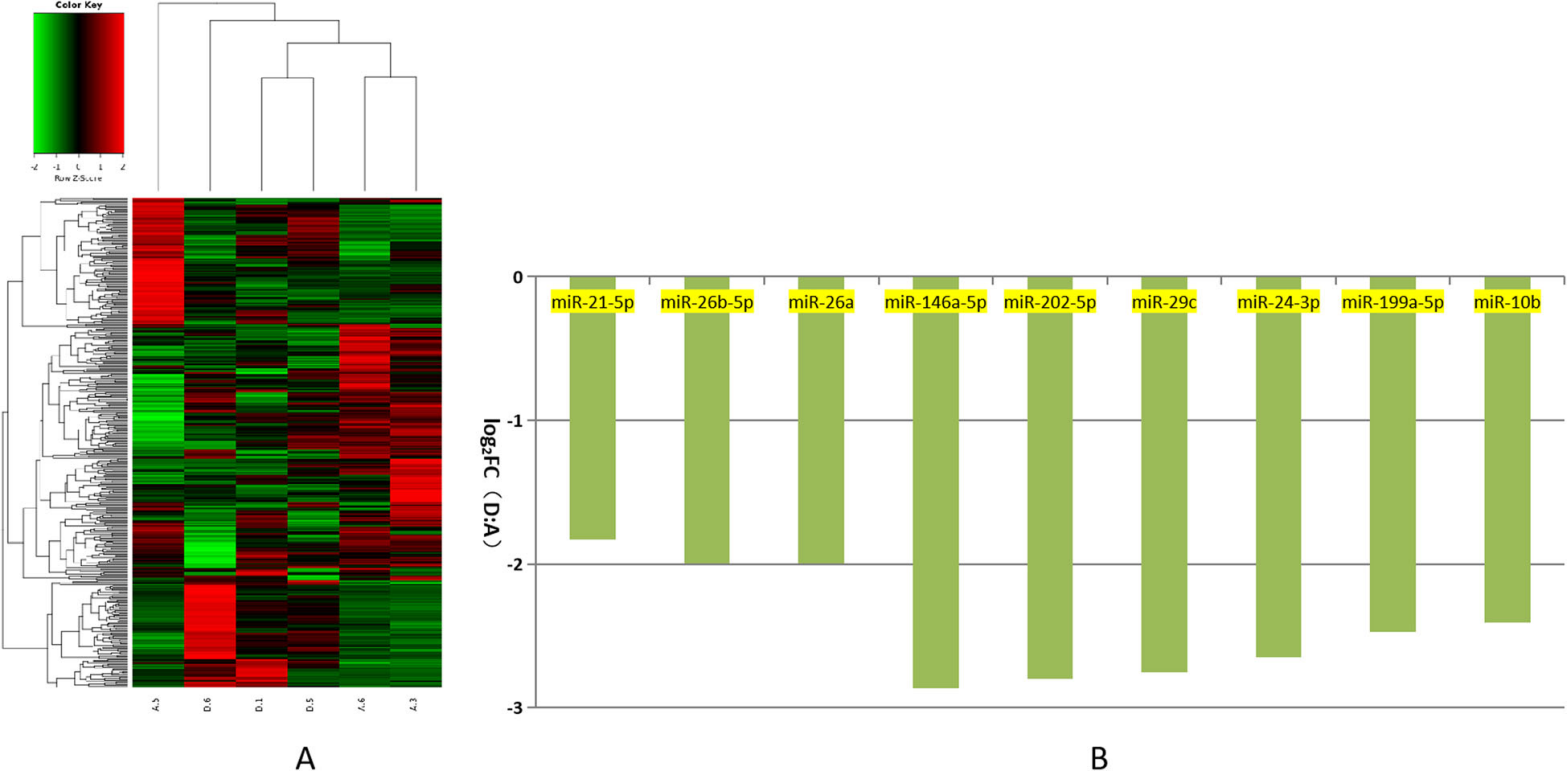
Comparison of most differentially expressed miRNAs between groups A and D. **a**. Heatmap of clustered genes indicated significantly different between groups A and D. **b**. Most significantly expressed miRNA genes between groups A and D. Y-axis indicates log2(Fold Changes, FC) of miRNA expression of group D versus A

These differentially expressed miRNAs were further analyzed using hierarchical clustering methods to form the heatmap comparing groups B to A (small size follicles: control group; Fig. 2a). As shown in Fig. 2b, we chose significant, differentially expressed miRNAs from group A—miR-10a-5p, miR-200b, miR-429, miR-192, miR-141, miR-221-3p, miR-425-5p, miR-7-5p, and miR-92a—and compared these to the control. These differentially expressed miRNAs were further analyzed using hierarchical clustering methods to form the heatmap comparing groups C to A (control group; Fig. 3a). As shown in Fig. 3b, we chose significant, differentially expressed miRNAs from group A—miR-194a-5p, miR-7137-3p, miR-182, miR-146b, miR-4332 and miR-9793-5p—and compared these to the control. Third, these differentially expressed miRNAs were further analyzed using hierarchical clustering methods to form the heatmap comparing groups D to A (control group; Fig. 4a). As shown in Fig. 4b, we chose significant, differentially expressed miRNAs from group A—miR-21-5p, miR-26b-5p, miR-26a, miR-146a-5p, miR-202-5p, miR-29c, miR-24-3p, miR-199a-5p, and miR-10b—and compared these to the control.

**Fig. 5:**
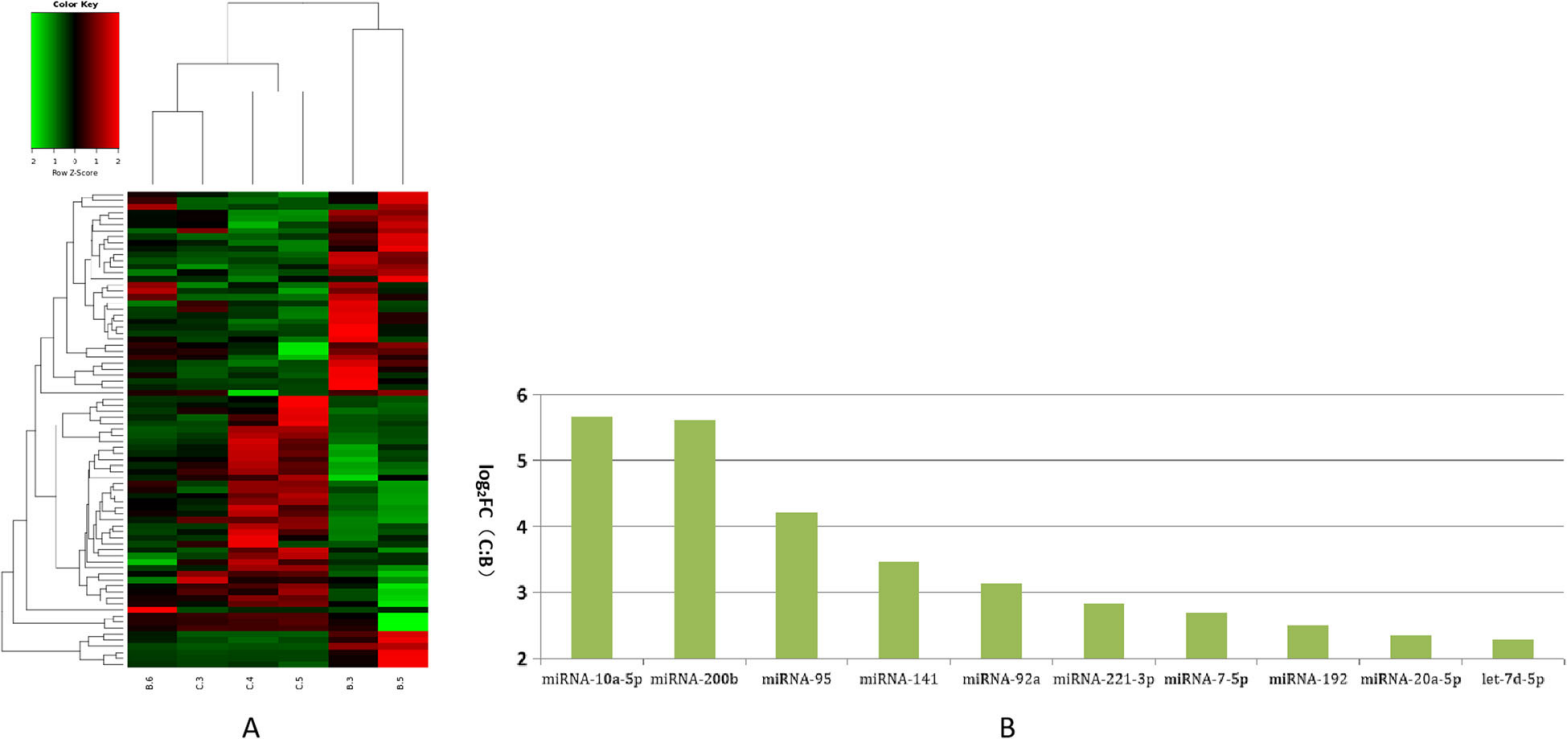
Comparison of most differentially expressed miRNAs between groups B and C. **a**. Heatmap of clustered genes between groups B and C. **b**. Most significantly expressed miRNA genes between groups B and C. Y-axis indicates log2(Fold Changes, FC) of miRNA expression of group C versus B

**Fig. 6:**
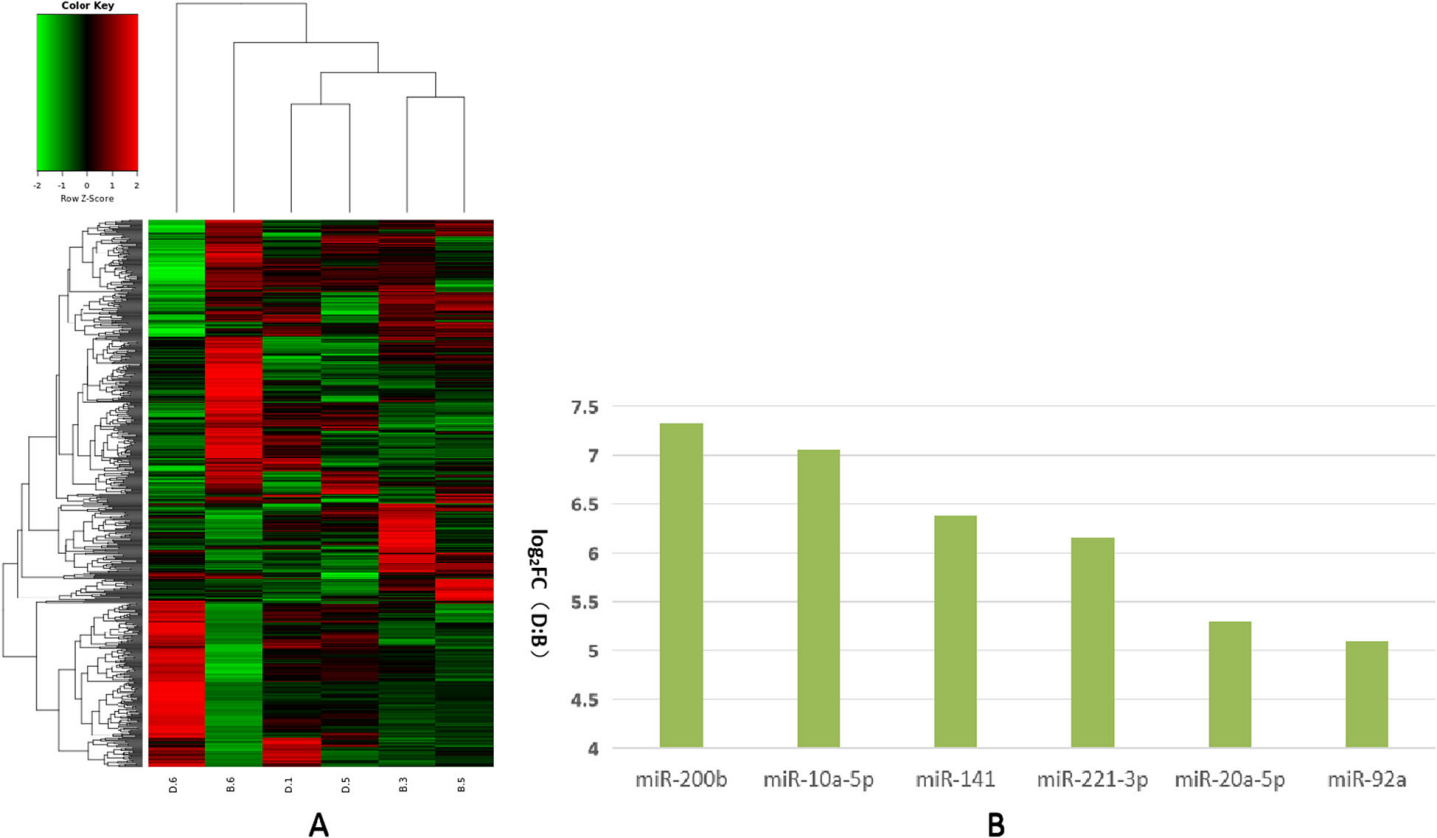
Comparison of most differentially expressed miRNAs between groups B and D. **a**. Heatmap of clustered genes indicated significantly different between groups B and D. **b**. Most significantly expressed miRNA genes between groups B and D. Y-axis indicates log2(Fold Changes, FC) of miRNA expression of group D versus B

**Fig. 7:**
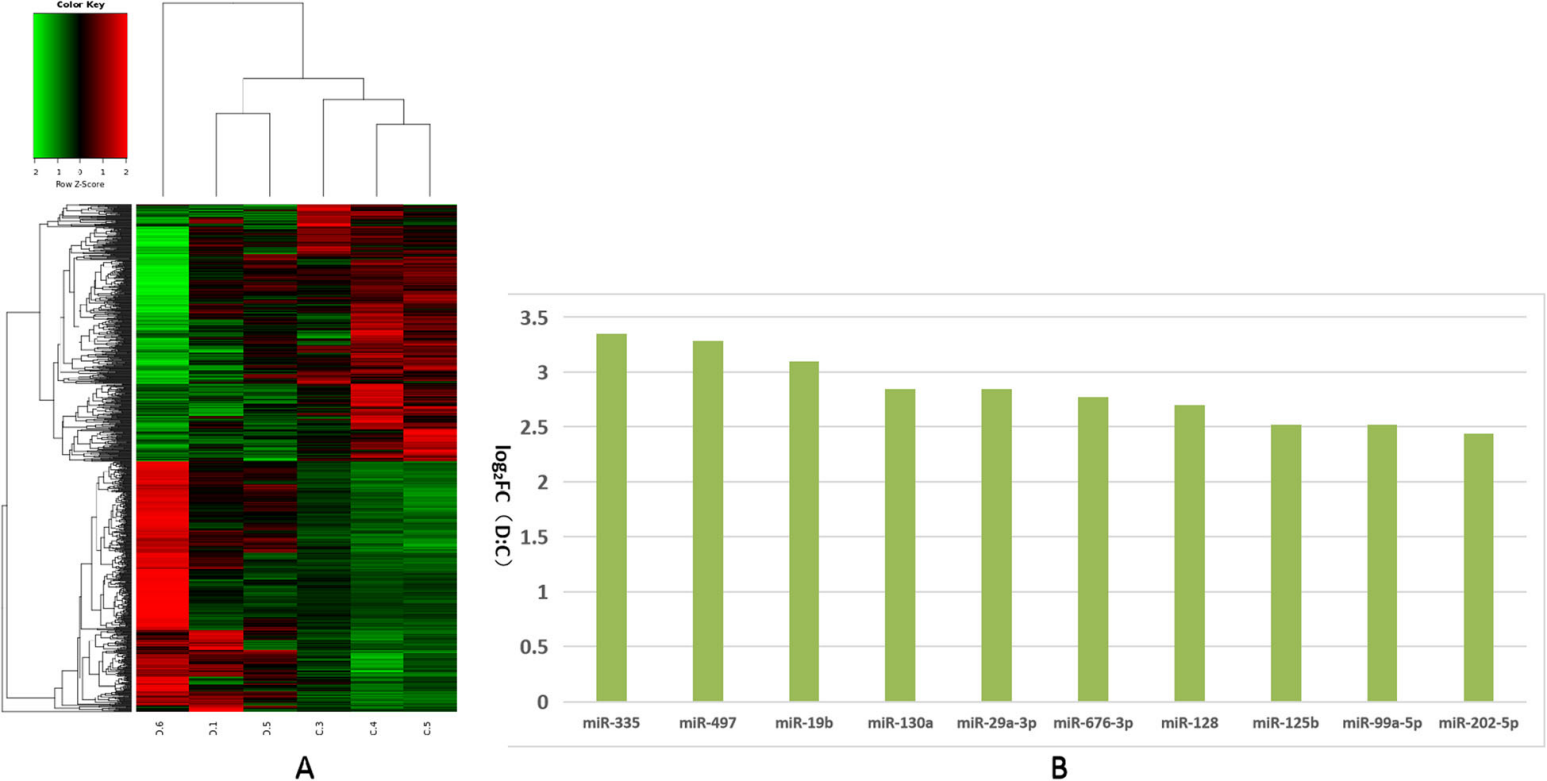
Comparison of most differentially expressed miRNAs between groups C and D. **a**. Heatmap of clustered genes shows significantly different between groups C and D. **b**. Most significantly expressed miRNA genes between groups C and D. Y-axis indicates log2(Fold Changes, FC) of miRNA expression of group D versus C

We further analyzed these differentially expressed miRNAs using hierarchical clustering methods to form the heatmap comparing groups B to C (Fig. 5a). We chose significant, differentially expressed miRNAs—miRNA-10a-5p, miRNA-200b, miRNA-95, miRNA-141, miRNA-92a, miRNA-221-3p, miRNA-7-5p, miRNA-192, miRNA-20a-5p, and let-7d-5p—from group B and compared them to group C (Fig. 5b). These differentially expressed miRNAs were further analyzed using hierarchical clustering methods to form the heatmap comparing groups B to D (Fig. 6a). We chose significant, differentially expressed miRNAs—miR-200b, miR-10a-5p, miR-141, miR-221-3p, miR-20a-5p, and miR-92a—of group B and compared these to group D (Fig. 6b). Finally, we further analyzed these differentially expressed miRNAs using hierarchical clustering methods to form the heatmap between groups C compared to group D (Fig. 7a). We chose significant, differentially expressed miRNAs—miR-335, miR-497, miR-19b, miR-130a, miR-29a-3p, miR-676-3p, miR-128, miR-125b, miR-99a-5p, and miR-202-5p—from group C and compared them to group D (Fig. 7b).

### GO and pathway analysis for miRNA target genes

Based on the above research results, we selected ten miRNAs among these four tested groups—miR-125b, let-7d-5p, miR-200b, miR-26a, miR-92a, miR-221-3p, miR-21-5p, miR-141, miR-99a-5p, and miR-10a-5p—as being related to porcine oocyte maturation (Figs. 2, 3, 4, 5, 6 and 7). These selected miRNAs were then used with Miranda and RNA software to predict the targeting regulation relationship between the whole species of miRNAs. This was done by taking the intersection results of the two prediction software programs as the final target gene prediction results.

**Fig. 8:**
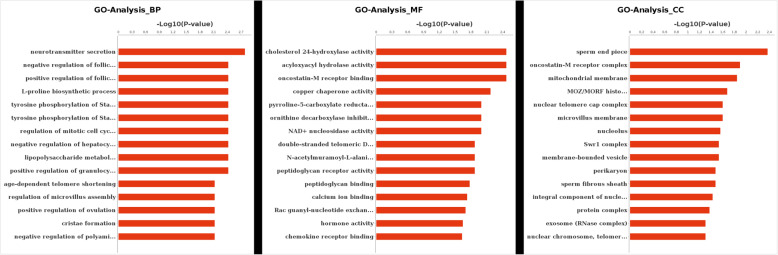
GO analysis indicates primary, differentially expressed miRNAs among the four groups. GO analysis shows results of all differentially expressed genes, which includes Biological Process (BP; left-most), Molecular Function (MF; middle), and Cellular Component (CC; right-most) in this figure. The coordinate axis Y axis is the Go-Term entry name, while the Coordinate axis X axis is log10 (*P*-Value). Red indicates significance, while blue indicates non-significance

**Fig. 9:**
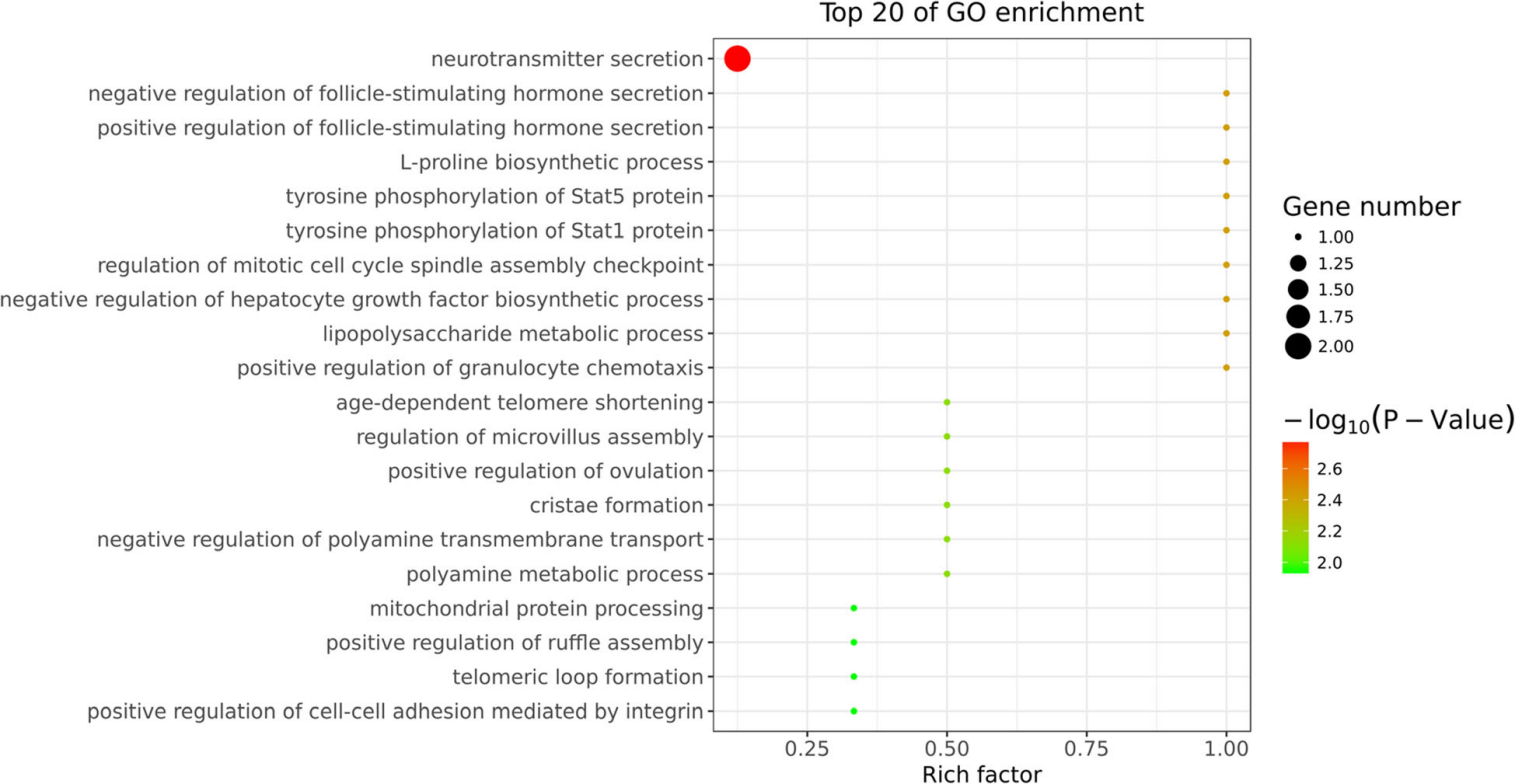
Ten miRNAs selected from different experimental groups were compared for functional analysis of target genes that are involved in reproductive development functional analysis. This image shows the first 20 entries into the Pathway-Term. The Y-axis is the value of Enrichment, while the X-axis is the Pathway-Term entry name. Red indicates significance, while blue indicates non-significance

Using this approach, miR-125b, let-7d-5p, and miR-200b were among the top 10 most highly expressed miRNAs from the four tested groups. This is sound evidence that these miRNAs have potential roles in regulating porcine oocyte development [20]. The parameters of this analysis were set as follows: energy_miranda<− 20, score_miranda> 150, energy_RNAhybird <− 25. Our results showed 67 significant, differentially targeted genes that were a hybrid between the Miranda and RNA software results. These results were then used to take GO further (Fig. 8) and to also take the pathway analysis further (Fig. 9).

**Fig. 10:**
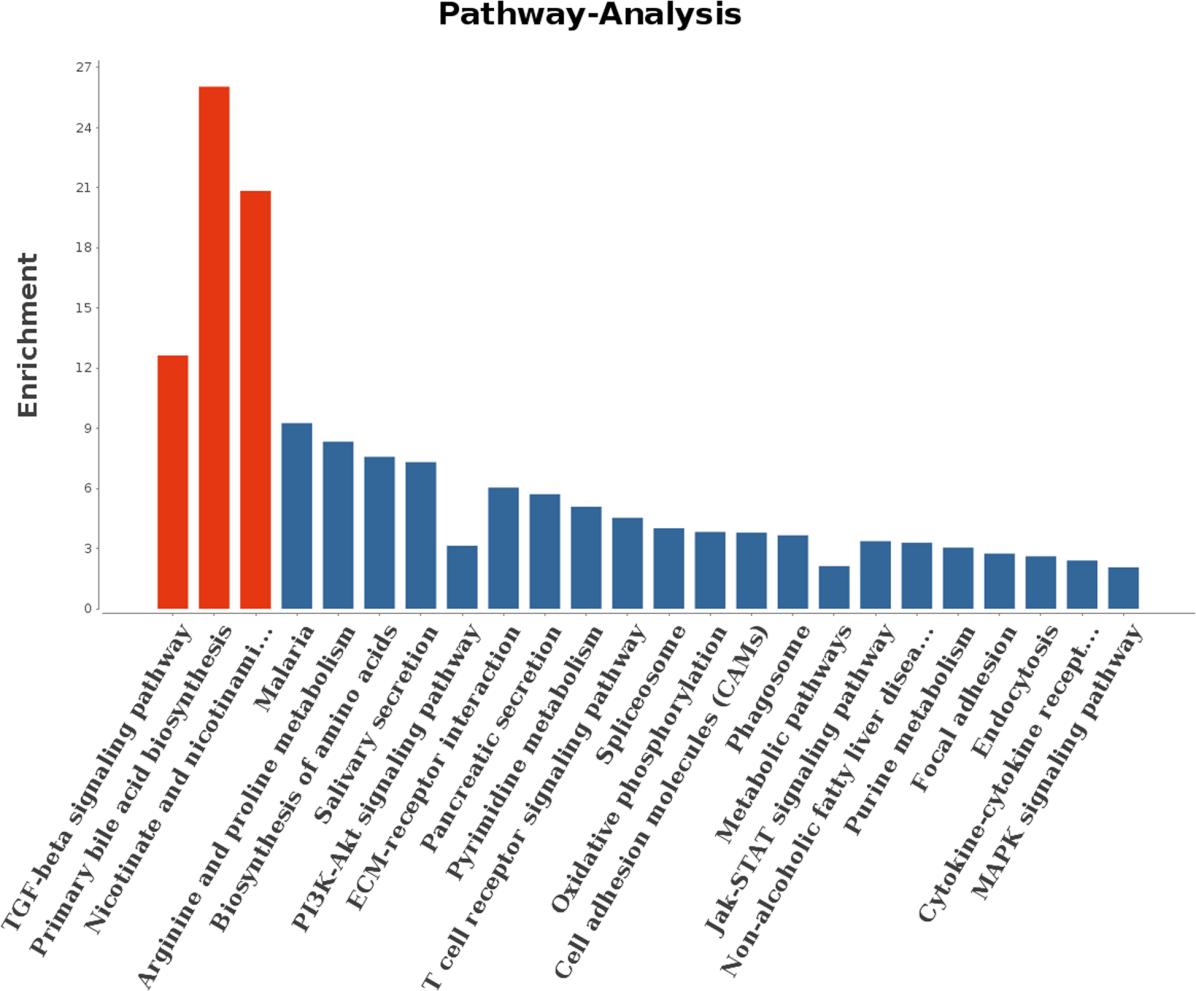
Functional analysis using GO enrichment scatter plot for 10 selected miRNAs targeting genes. The scatter plot is a graphical display of our GO enrichment analysis results. The degree of GO enrichment in the figure is measured by Rich factor, *P*_value, and the number of genes enriched on this pathway. The Rich factor refers to the ratio of the number of differentially enriched genes in the GO analysis to the number of annotated genes. The larger the Rich factor, the greater the degree of enrichment

**Fig. 11:**
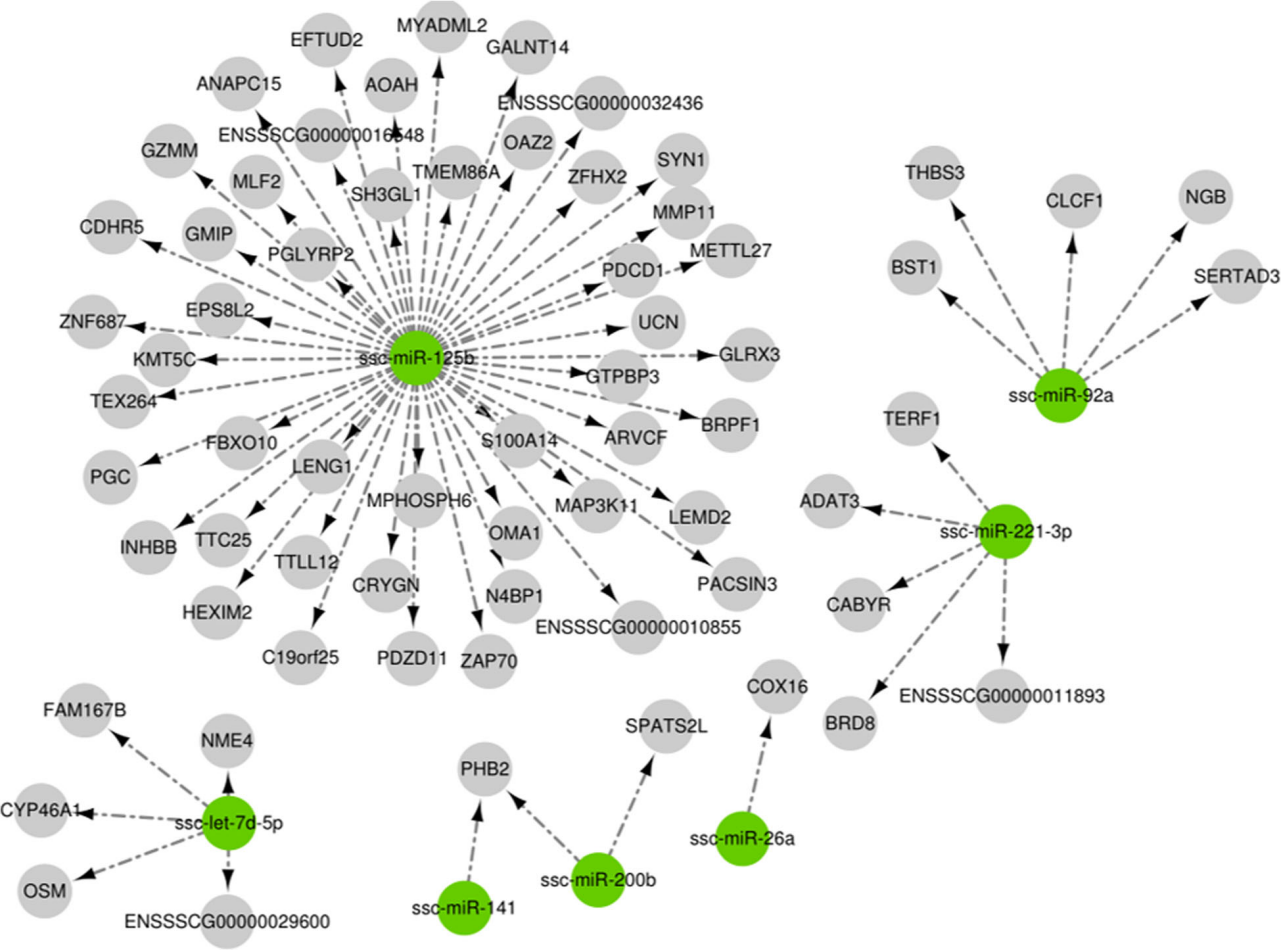
Functional map of target gene interactions among 7 miRNAs selected from experimental groups. Seven different miRNA (green color) and their targeting mRNAs (gray color) are sorted by color

These results showed that the gene number and significance to neurotransmitter secretion changed greatly across the GO enrichment scatter plot (Fig. 9). These results also show that the rich factor value of the regulation of follicle-stimulating hormone (FSH) secretion was almost 1 (Fig. 10). This indicated the functions of many targeting gene by these miRNAs were primarily focused on oocyte development by FSH simulating porcine follicular growth. As presented in the map in Fig. 11, we further clarified the interaction of these targeting mRNAs and miRNAs genes (miR-125b, let-7d-5p, miR-200b, miR-26a, miR-92a and miR-221-3p). As indicated, these indicated genes were all closely related to reproductive system function.

## Discussion

As is already known, ovarian follicles provide a unique microenvironment for oocyte development and allow for interactions between follicular somatic cells and oocytes. Given this, it is crucial to study the components of follicular fluid to better elucidate their role in the mechanism of oocyte maturation. Previous work has shown that exosomes (EXs) are an essential carrier for the signal transduction occurring between follicular somatic cells and oocytes in follicular fluid [21, 22]. Here, PFF exosomes were successfully isolated and confirmed using transmission electron microscopy. Our findings were similar to those of other reports [23].

To better understand the molecular mechanism behind oocyte maturation, it is crucial to reveal the axis of follicular somatic cells-EXs-oocyte by studying the expressed genes in the PFF EXs at the level of the RNA. Past work has used bioinformatics analysis to identify potential miRNA targets which were very important to growth of porcine oocytes in follicles [24, 25]. In particular, follicle size has been shown to be closely related to oocyte development [26, 27].

Mature miRNAs are produced by a series of nuclease-mediated cleavage processes of longer primary transcripts. These miRNAs are then assembled into RNA-induced silencing complexes to identify target genes, which is done by base complementary base pairing. The degree of complementarity guides the silencing complexes to their target genes, whereby they degrade or block the translation of their target genes. Given this, we conducted an miRNA sequencing experiment using a total of 12 PFF exosome samples (three repeats per group, with groups composed of different follicle sizes) according to previously described methods. First, our results showed that approximately 99% of all valid reads were with groups A-D. As indicated in Table 1, we successfully mapped 12 samples to the porcine non-coding RNA database for further analysis. Our results also showed that 8 miRNA genes obtained from PFF EXs (miR-10a-5p, miR-200b, miR-429, miR-192, miR-141, miR-221-3p, miR-425-5p, miR-7-5p and miR-92a) had significantly enhanced expression in porcine follicles with diameters < 3 mm to 5 mm. Moreover, miR-10a-5p expression was almost 8 times higher than that in group A (Fig. 2). According to these results, it can be indicated that miR-10a-5p mainly limited expressed in small follicles (contained immature oocytes), but these miRNAs (miR-10a-5p, miR-200b, miR-429, miR-192, miR-141, miR-221-3p, miR-425-5p, miR-7-5p and miR-92a) enhanced the expression with follicle growth (from 5 mm diameter follicles to previous ovulation stage), which is very important to elucidate the mechanism of porcine oocyte maturation.

Our results also indicated that miR-10a family expression was closely related to reproductive system function. More specifically, miR-10a and miR-10b were both expressed at basal levels in granulosa cells, but were highly expressed in theca and stroma cells within the ovary. This indicated they could repress proliferation and induce apoptosis in human, mouse, and rat granulosa cells. This would partly occur by BDNF repression through direct binding to its 3′ UTR, with the miR-10 family and the TGF-β pathway forming a negative feedback loop in GC^s^ [28]. With porcine follicles of larger diameter (5–8 mm), there was significantly enhanced expression of six miRNA genes obtained from PFF EXs (miR-194a-5p, miR-7137-3p,miR-182, miR-146b, miR-4332 and miR-9793-5p) when compared to the control group (< 3 mm). Of these, miR-194a-5p expression was almost four times higher than that of group A (Fig. 3). Exosomes act as carriers to follicles from the surrounding somatic cells as well as the body and blood to influence oocyte maturation. Moreover, immune stimulus has been shown to enhance vasodilatation to promote exosomal function [29, 30]. Past work has also shown that miR-194a family expression plays an important role in immunological responses and function, including the flounder [31] and zebrafish [32] immune responses. Further results shown that miR-194a-5p is highly related to oocyte maturation of follicles growing stage, which can induce immune reaction to enhance ovary local blood circulation to provide more nutrients.

When follicles grow to a diameter of 8 mm, 9 miRNA genes obtained from PFF EXs (miR-202-5p, miR-199a-5p, miR-21-5p, miR-26b-5p, miR-26a, miR-29c, miR-24-3p, miR-146a-5p, and miR-10b) had significantly reduced expression when compared to the control group (< 3 mm). Of these, both miR-46a-5p and miR-202a-5p gene expression levels were almost three times lower than those of group A (Fig. 4). It has been shown that the miR-202a-5p gene is highly expressed in bovine un-maturated oocytes [33], while miR-199a-5p gene is highly expressed in germinal vesicle stage occytes [34]. From groups B, C, and D, these selected, differentially expressed miRNAs were all clustered in Figs. 5, 6, and 7 for further analysis. This analysis confirmed earlier work, indicating that some were closely related to oocyte development [35]. Based on the significant expression of the above miRNAs, we selected ten relevant miRNAs—miR-10a-5 ps, miR-200b, miR-141, miR-92a, miR-221-3p, miR-21-5p, miR-26a, let-7d-5p, miR-125b and miR-99a-5p—related to porcine oocyte development for further target genes analysis. Currently, there is no gold standard for miRNA gene target analysis [36, 37]. Given this, we used Miranda and RNA software to predict the targeting regulation for further GO and pathway analyses according to previously reported papers [38, 39].

This analysis found selected miRNAs (miR-125b, let-7d-5p, miR-200b, miR-26a, and miR-92a) were expressed in PFF exosomes obtained from different-sized follicles. These miRNAs were also very closely related to oocyte development, which has been previously reported [40]. The gene number and their significance regarding neurotransmitter secretion changed greatly (Figs. 8 and 9). It is known that the activity of the hypothalamus-pituitary-ovarian endocrine axis is very vigorous during processes related to porcine follicular growth [41]. These results also show that many targeted genes were involved in the regulation of FSH secretion (Figs. 7 and 8), This corroborates previous work, which has shown how FSH controls the follicular development in different-sized follicles [42, 43]. Therefore, we concluded that the functions of many of these miRNA-targeted genes were primarily focused on oocyte development by FSH simulation. This effect occurs through the porcine follicular growth stage, from its early (group A), middle (group B), and matured (groups C and D) stages. Collectively, these results showed that important pathways identified from this work included those related to the biosynthesis of TGF-beta signaling, primary bile acid biosynthesis, and both nicotinate and nicotinamides metabolism (Fig. 10). Importantly, these potential target genes are all closely related to oocyte development and follicle growth. Past work has also shown that many of these miRNA-targeted genes were mainly involved in TGF-beta-related signaling. This pathway may play a significant role during the early stages of porcine oocytes nuclear and cytoplasmic maturation.

It should be clarified that while these are potential genes predicted from our GO and pathway analyses, their associations as observed in previous published papers are limited. Some past work has shown that each miRNA can have many gene targets and that these gene targets may be different and based on cell type [44]. Moreover, the exact influence of one miRNA on gene expression may be physiologically indispensable, but difficult to identify statistically. In conclusion, this study provides new insights into the global transcriptome changes and the abundance of specific transcripts in porcine oocytes that have been correlated with follicle size.

Above all, our study has identified differently expressed miRNAs obtained from PFF EXs in different-sized follicles (groups B, C, and D) when compared to the control (group A). Using our GO and pathway analyses, we have identified different targeting cluster genes—miR-125b, let-7d-5p, miR-200b, miR-26a and miR-92a—important to porcine oocyte maturation. These miRNAs could be used as biomarkers for a better understanding of the oocyte maturation process and allow the selection of high-quality oocytes for further research. Despite this work, there remain many open questions about porcine oocyte development mechanisms related to exosomal miRNAs. For instance, how do these miRNAs control the mechanisms regarding oocyte cytoplasmic and nuclear maturation?

## Conclusions

This work first showed miRNAs obtained from PFF exosomes of different-sized follicles had significantly differential expression relative to small-sized follicles. We also identified some critical miRNAs genes—miR-125b, let-7d-5p, miR-200b, miR-26a, and miR-92a—that may be potential biomarkers for the molecular identification of high-quality oocytes. These miRNAs may also serve as future identifiers for the mechanism behind oocyte maturation. Despite this work, research into miRNAs and porcine oocyte maturation still has many open questions that need to be answered to fully understand the molecular mechanisms behind this complicated process.

## Data Availability

The datasets during and/or analyzed during the current study available from the corresponding author on reasonable request.
